# Parenthood for childhood cancer survivors: unfounded fear of cancer development in offspring and related health behaviors

**DOI:** 10.3389/fpsyg.2023.1269216

**Published:** 2024-01-11

**Authors:** Nina Dalkner, Eva Fleischmann, Anja Borgmann-Staudt, Christine Fürschuß, Stephanie Klco-Brosius, Katerina Kepakova, Jarmila Kruseova, Herwig Lackner, Gisela Michel, Andrea Mohapp, Eva Nagele, Anna Panasiuk, Melanie Tamesberger, Eva Z. Reininghaus, Karin Wiegele, Magdalena Balcerek

**Affiliations:** ^1^Department of Psychiatry and Psychotherapeutic Medicine, Medical University of Graz, Graz, Austria; ^2^Department of Pediatric Oncology and Hematology, Charité Berlin, Berlin, Germany; ^3^Division of Paediatric Haematology/Oncology, Medical University of Graz, Graz, Austria; ^4^Czech Republic and International Clinical Research Center (FNUSA-ICRC), University Hospital Brno, Brno, Czechia; ^5^Department of Pediatric Haematology and Oncology, University Hospital Motol Prague, Prague, Czechia; ^6^Faculty of Health Sciences and Medicine, University of Lucerne, Lucerne, Switzerland; ^7^Bone Marrow Transplantation Unit, Medical University of Wroclaw, Wrocław, Poland; ^8^Department of Pediatric Oncology, Kepler Universitätsklinikum, Linz, Austria; ^9^Berlin Institute of Health, Berlin, Germany

**Keywords:** fear, offspring health, childhood cancer, survivor, health-related behavior

## Abstract

Current literature reveals no increased risk for adverse non-hereditary health outcomes in the offspring of childhood cancer survivors (CCS), yet survivors reported concerns regarding their offspring’s health. To investigate how the fear of cancer development in offspring influences parental behavior related to health and prevention, survey reports from 256 European adult CCS and 256 age- and sex-matched siblings who participated in a multicenter study on offspring health were analyzed in the present study. Analyses of covariance and chi-square tests were conducted to test for differences between CCS and siblings in outcome variables (all related to healthy parenting behavior). CCS reported higher fear levels (*p* = 0.044, Partial η^2^ = 0.01) and less alcohol consumption (*p* = 0.011, Phi = 0.12) and smoking (*p* = 0.022, Phi = 0.11) during pregnancy than siblings. In survivor families, children were breastfed less often (*p* < 0.001, Phi = 0.18). Partial correlation analyses showed that CCS’ fear levels decreased with increasing age (*r* = −0.16, *p* = 0.014), time since oncological therapy (*r* = −0.19, *p* = 0.003), and number of children (*r* = −0.21, *p* = 0.001). Overall, due to their own experiences with cancer, many CCS harbor misperceptions regarding the health outcomes of their offspring. Although the fear decreases with increasing distance from the active disease, any fear should be taken seriously, even if unfounded, and combated through targeted educational measures.

## 1 Introduction

Childhood cancer survival rates have increased considerably over the past few decades, and the probability of reaching adulthood is now at 80% in developed European countries (Winther et al., 2015; Ferrari and Barr, 2017). Nonetheless, childhood cancer survivors (CCS) are a particularly psychologically vulnerable group, often exhibiting psychological distress during long-term follow-up. A recent meta-analysis in adolescent and young adult cancer survivors from Europe and North America revealed a higher occurrence of psychiatric diagnoses including mood and anxiety disorders compared to cancer-free controls (De et al., 2020). In this context, and especially among women (Michel et al., 2010), a significant proportion of CCS in comparison to the general population (Brinkman et al., 2013) has reported heightened levels of anxiety, depressive symptoms, and somatization—the manifestation of mental states as physical symptoms. The level of psychological distress in CCS is associated with a variety of risk factors, including a low educational level, an unmarried status, an annual household income < $20,000, unemployment, and lack of health insurance (Zeltzer et al., 2008). The recognition and alleviation of psychological distress is an often, unmet need in long-term follow-up and must be improved (Michel et al., 2010).

Although mental health problems are frequent in CCS (Friend et al., 2018), there is no higher risk for substance use disorder (De et al., 2020). In contrast, compared to their siblings and peers, CCS exhibit similar or even lower health risk behaviors, such as smoking, binge drinking, and drug use (Klosky et al., 2012; Marjerrison et al., 2016). In fact, a cancer diagnosis often leads to changes in prevention-oriented health behaviors (Ford et al., 2014), possibly due to CCS’s higher awareness of their own vulnerability (Hawkins et al., 2010). To cope with experiences related to the lifelong impact of cancer and to maintain a positive attitude, CCS have developed a variety of strategies (Belpame et al., 2021) and resilience in their present lives (Hinton et al., 2022), as well as post-traumatic growth (Tremolada et al., 2016).

Starting a family is an important step for many and is accompanied by numerous challenges. In previous studies, CCS considered offspring health as an issue of great concern (Balcerek et al., 2015). Consequently, CCS expressed concern that that the health of their future children could be negatively affected by their own previous cancer disease and treatment, or that their offspring could also develop cancer (Winther et al., 2004; Tawn et al., 2005; Morton et al., 2017).

However, according to current literature, there is no indication of an increased risk for non-hereditary cancers. Studies from Scandinavia and the USA have consistently demonstrated no elevated risk concerning genetic instabilities, malformations, or non-hereditary cancer in CCS offspring (Sankila et al., 1998; Winther et al., 2004, 2008, 2010; Nagarajan and Robison, 2005; Tawn et al., 2005; Rees et al., 2006; Signorello et al., 2012). These results are based on studies using information from a cancer registry (5.874 offspring, 22), hospital registry data (1.715 offspring, 23), and a cytogenetic registry (2,630 offspring, 18). Accordingly, an U.S. American CCS Study, which examined 4.699 offspring from 2.755 CCS regarding congenital anomalies, revealed no increased risk for malformations in offspring whose parents had received mutagenic therapies (Signorello et al., 2012). Overall, CCŚ offspring were found to be as healthy as their peers and did not exhibit an elevated risk of developing congenital anomalies compared to their counterparts, even after Assisted Reproductive Technology (ART; Sommerhäuser et al., 2021; Borgmann-Staudt et al., 2022). While Ripperger et al. (2017) described a cancer predisposition syndrome, and familial immunodeficiency can increase the risk of childhood cancer in some cases (Armstrong et al., 2009), the influence of genetic variation in CCS is inconsistent in genome-wide association studies (Clemens et al., 2018). Much of the heredity of a potential cancer predisposition remains unexplained. In addition, lifestyle risk factors do not seem to play a major role in a child’s risk for cancer (Spector et al., 2015). Furthermore, it was recently shown that CCS offspring had comparable to increased health-related quality of life compared to their peers (Balcerek et al., 2021). Despite these recent findings, CCS may not be aware of these positive outcomes. Consequently, they may have developed unfounded fears, which potentially affect health- and prevention-related parental behaviors. In the Multicenter Offspring Study, we therefore explored health- and prevention-related parental behavior of CCS and a sibling control group linked to the fear of cancer development or adverse health outcomes in their children. This specific aspect has not been previously addressed in current literature. We hypothesized that CCS would report higher levels of fear of cancer development in their offspring compared to a group of siblings of CCS. Consequently, we further hypothesized that the level of fear would show a positive correlation with preventive and favorable health and parenting behaviors, and that the strength of the correlations would differ between CCS and the siblings group. We additionally hypothesized that survivors from cancer types with higher rates of mortality, such as central nervous system tumors, bone tumors, soft tissue tumors, and acute myeloid leukemia, would be more fearful and exhibit different parenting behavior than survivors from cancer types with better prognosis, including leukemia, lymphoma, retinoblastomas, and Wilms tumors (National Cancer Registration and Analysis Service, 2021; National Cancer Institute, 2023).

Based on these hypotheses, the following research questions were addressed by the current study:

1. Is there a difference in outcome variables (a. fear that offspring might develop cancer, b. favorable/unfavorable health behaviors) in CCS compared to siblings?
2. Is the level of fear that offspring might develop cancer associated with favorable and unfavorable health behaviors in CCS compared to a group of siblings?
3. Do CCS and siblings differ in the associations between the fear that offspring might develop cancer and favorable vs. unfavorable health behaviors?
4. Are the types of cancer (hematological tumors vs. brain tumors vs. other solid tumors), the age at treatment, and the time since treatment associated with the level of fear that offspring might develop cancer?

## 2 Materials and methods

### 2.1 Participants and procedure

The Multicenter Offspring Study surveyed CCS and siblings between March 2013 and December 2016. Paper-based questionnaires were distributed to former patients who had been treated in pediatric oncology centers in Austria, the Czech Republic, Germany, Poland, and Switzerland. Inclusion criteria required participants to be 18 years or older and to have had at least one child. CCS must have received treatment in one of the participating centers, and their offspring must have been born after treatment. Detailed information on the study methods is available in our previous report on the Multicenter Offspring Study (Balcerek et al., 2015).

Overall, 2.221 individuals participated in the survey (1.126 CCS with 1.780 offspring and 271 siblings with 441 offspring). Data were collected in 5 European countries including 384 German (75%), 26 Austrian (5.1%), 57 Czech (11.1%), 4 Polish (0.8%), and 41 Swiss participants (8.0%).

The overall mean response rate was 59.7% with varying rates across countries (Austria: 85.0%, Czech: 32.1%, Germany: 65.8%, Poland: total number of CCS and siblings contacted not available, Switzerland: 37.2%). However, the number of recruited siblings (*n* = 271) was relatively low in comparison to the number of CCS (*n* = 1.780). To ensure an age- and sex-matched group, we undertook matching from the pool of siblings, which resulted in the proportion of remaining participants from Germany being the highest.

In total, for the present analyses, full data sets of 512 age- and sex matched CCS and siblings (256 participants in each group) were used after the exclusion of 15 siblings with incomplete datasets. Participants had given informed consent prior to participation, and the survey was approved by the lead study center ethics committee (Charité-Universitätsmedizin Berlin; EA2/237/05, EA2/103/11) and the respective ethical boards of the cooperating centers in accordance with the Declaration of Helsinki, ICH guideline for Good Clinical Practice and current regulations.

### 2.2 Outcome variables

Questionnaires were conducted in corresponding languages. Both layout and items of the questionnaire were based on the Robert Koch Institute’s health survey (KIGGS study), conducted 2003–2006 in a large cohort of children and adolescents from the German general population (Hölling et al., 2012). Our study questionnaires were tailored to explore health aspects in CCS offspring, including non-previously validated questions, as these have not yet been previously addressed. In the current study, broad demographic, and clinical characteristics of participants, as well as information concerning the fear of possible cancer development in offspring and related health behaviors were assessed.

To assess participant fear, the following question was presented: “How worried are you that your child might develop cancer?” by marking their fear on a 10 cm long visual scale from 0 to 10, with 0 = little to no fear and 10 = highly fearful. The position of the marking was then measured starting at 0, and fear level corresponded to the number of cm on the scale. According to a previously published paper including this variable, fear was also categorized into three categories: none/low (< 2 cm), medium (2–6 cm), and high/very high [> 6 cm; (Balcerek et al., 2021)].

Favorable and unfavorable health-related behaviors included the questions, “Did the child’s mother smoke/consume alcohol during pregnancy?,” “How many months was the child breastfed?,” and “Do you smoke in the presence of your child at home?.” Three of the questions were answered with yes or no, and in the case of breastfeeding, duration was specified in months. We used all dichotomous variables to form a risk behavior score ranging from 0¬ to 4, with a higher score indicating higher risk behavior. A detailed description of the risk behavior score is displayed on Table 1.

**Table 1:** Types of health-related behaviors and their methods of operationalization administered to a sample of 256 childhood cancer-survivors and 256 age- and sex-matched siblings.

Type of behavior	Variable	Question	Operationalization
Unfavorable	Alcohol consumption during pregnancy*	*“Did the child’s mother consume alcohol during pregnancy?”*	Yes = 1 No = 0
	Smoking during pregnancy*	*“Did the child’s mother smoke during pregnancy?”*	Yes = 1 No = 0
	Smoking in child’s presence*	*“Do you smoke in the presence of your child at home?”*	Yes = 1 No = 0
Favorable	Breastfeeding*	*“How many months was the child breastfed?”*	≥ 6 ➔ 0 < 6 ➔ 1
	Vaccinations	Number of total vaccinations^a^	Number
	Doctor appointments	Number of doctor appointments in the last 12 months	Number

*The four dichotomous variables were used to create the risk behavior score ranging from 0 to 4, with a greater number indicating higher risk behavior.

a Vaccinations: measles, mumps, rubella, diphtheria, tetanus, pertussis, poliomyelitis, hepatitis B, and Haemophilus influenzae type B.

In addition to the risk behavior score, the number of doctor appointments attended in the last 12 months and the total number of vaccinations were considered as favorable and/or preventive health behaviors. Vaccinations included measles, mumps, rubella, diphtheria, tetanus, pertussis, poliomyelitis, hepatitis B, and *Haemophilus influenzae* type B—all of which were free of charge in participating countries.

### 2.3 Data analysis

Study data was analyzed for two groups (CCS vs. an age- and sex-matched sibling control group, 1:1 matching). Chi-square and Student’s t-tests were conducted to compare demographic characteristics between siblings and CCS (age and sex of parent and child, highest vocational education in household, country, number of children, age when treated, type of cancer). Shapiro–Wilk tests showed a lack of normal distribution in several variables, however, as the sample size is n > 30, normality can be assumed (Kwak and Kim, 2017).

To compare fear level and health behaviors (number of doctor appointments and vaccinations, risk behavior score, breastfeeding duration) between siblings and CCS, chi-square tests and analyses of covariance (ANCOVAs) adjusting for age and sex of parent and child as well as country and highest vocational education in household were calculated. Levene’s test was significant when comparing fear level and the number of vaccinations. A multivariate ANCOVA (MANCOVA) could not be calculated due to the Box’s M test being significant. This was the case as well when comparing different cancer types (hematologic tumors, brain tumors, and other solid tumors) in CCS regarding fear level and health behaviors.

Partial correlation analyses to determine the associations between fear level and the number of doctor appointments, the number of vaccinations, the time since oncological therapy, and the risk behavior score with the covariates age and sex of parent and child, country, highest vocational education in household, and type of cancer for CCS were conducted. Further correlations between fear level and the age of parent and child as well as the parent’s age when treated in CCS with the covariates sex of parent and child, highest vocational education in household, and type of cancer for CCS were calculated. False discovery rate (FDR) was used to adjust for multiple testing, and Fisher’s r-to-z transformation was calculated to compare the correlations of CCS and siblings (Weiss, 2011).

## 3 Results

### 3.1 Sociodemographic characteristics

The final sample consisted of 512 age- and sex-matched participants (256 CCS, 256 siblings). None of the offspring in either cohort had developed cancer at the time of survey. Participants differed in their household’s highest level of vocational education and home country (see Table 2): CCS did not have any professional degree or were still in training more often than siblings, who had a higher percentage of polytechnic or university degree, albeit with small effect size. CCS were more often German, whereas siblings were more often Czech. Both groups were similar in offspring number as well as child’s age and sex.

**Table 2:** Sociodemographic information about age- and sex-matched CCS and their siblings (*n* = 512).

Variables	Groups				
	CCS (*n* = 256)	Siblings (*n* = 256)	Statistics	*p*	Effect size
Age* (M ± SD)	38.5 (6.0)	38.7 (6.6)	*t* (510) = −0.28	0.784	Cohen’s d = −0.02
Sex*					
Female	169 (66.0%)	169 (66.0%)	χ^2^(1) = 0.00	1.000	Phi = 0.00
Male	87 (34.0%)	87 (34.0%)			
Country			χ^2^(1) = 25.75^a^	**<0.001**	Cramér’s V = 0.22
Austria	9 (3.5%)	17 (6.6%)			
Czech Republic	13 (5.1%)	44 (17.2%)			
Germany	212 (82.8%)	172 (67.2%)			
Poland	3 (1.2%)	1 (0.4%)			
Switzerland	19 (7.4%)	22 (8.6%)			
Highest vocational education in household			χ^2^(1) = 8.42^a^	**0.030**	Cramér’s V = 0.13
No professional degree / Still in training	4 (1.6%)	0 (0.0%)			
Apprenticeship / Vocational school	102 (39.8%)	85 (33.2%)			
Master / other technical school	46 (18.0%)	41 (16.0%)			
Polytechnic / University degree	104 (40.6%)	130 (50.8%)			
Number of biological children	1.96 (0.72)	1.95 (0.73)	t(510) = 0.18	0.855	Cohen’s d = 0.02
Child’s age (M ± SD)	8.20 (5.88)	8.29 (6.71)	*t* (501,335) = −0.17	0.867	Cohen’s d = −0.02
Child‘s sex					
Female	128 (50.0%)	121 (47.3%)	χ^2^(1) = 0.28	0.596	Phi = −0.03
Male	128 (50.0%)	135 (52.7%)			
Age when treated (*M* ± SD)	10.42 (4.48)				
Time since treatment (M ± SD)	28.10 (6.10)				
Cancer type					
Leukemia	115 (44.9%)				
Lymphoma	47 (18.4%)				
Bone tumors	29 (11.3%)				
Soft tissue tumors	21 (8.2%)				
Brain tumors	14 (5.5%)				
Neuroblastoma	9 (3.5%)				
Renal tumors	9 (3.5%)				
Germ cell tumors	9 (3.5%)				
Other malignant epithelial neoplasm	3 (1.2%)				

CCS, childhood cancer survivors; *matching variable.

a Two-tailed Fisher’s exact test was used. Significant *p* values are marked in bold.

### 3.2 Fear levels and health-related behaviors

Table 3 shows group differences in outcome variables that include fear of possible cancer development in offspring and favorable and unfavorable health behaviors. Five ANCOVAs with the covariates age and sex of parent and child, country, and highest vocational education in household showed that CCS had higher levels of fear and a lower risk behavior score, while the total number of vaccinations, doctor appointments, breast-feeding duration did not differ for their children. Chi-square tests showed that compared to siblings, CCS consumed less alcohol and smoked less during pregnancy; they also breastfed their children less often. However, there was no difference in the percentage of individuals who smoked at home in the presence of their child, and the tests revealed a similar breastfeeding duration of at least 6 months.

**Table 3:** Comparison of age- and sex-matched CCS and siblings in outcome variables pertaining to favorable/ preventive behavior and the fear of cancer development in their children (*n* = 512).

Variables	Groups				
	CCS (*n* = 256)	Siblings (*n* = 256)	Statistics	*p*	Effect size
Fear of possible cancer development in offspring^a^^,^^b^	4.10 (2.96)	3.52 (2.84)	*F* (1,504) = 4.08	**0.044**	Partial η^2^ = 0.01
None/low (< 2)	94 (36.7%)	109 (42.6%)	χ^2^(2) = 2.72	0.257	Cramér’s V
Medium (2–6)	94 (36.7%)	93 (36.3%)			= 0.07
High/very high (> 6)	68 (26.6%)	54 (21.1%)			
Alcohol consumption during pregnancy			χ^2^(1) = 6.40	**0.011**	Phi = 0.12
Yes	11 (4.3%)	27 (10.5%)			
No	245 (95.7%)	229 (89.5%)			
Smoked during pregnancy			χ^2^(1) = 5.26	**0.022**	Phi = 0.11
Yes	8 (3.1%)	21 (8.2%)			
No	248 (96.9%)	235 (91.8%)			
Smoked at home in child’s presence			χ^2^(1) = 0.67	0.412	Phi = 0.05
Yes	10 (3.9%)	15 (5.9%)			
No	246 (96.1%)	241 (94.1%)			
Breastfeeding			χ^2^(1) = 14.85	**< 0.001**	Phi = 0.18
Yes	202 (78.9%)	234 (91.4%)			
No	54 (21.1%)	22 (8.6%)			
Breastfeeding ≥ 6 months			χ^2^(1) = 2.04	0.153	Phi = 0.07
Yes	119 (46.5%)	102 (39.8%)			
No	137 (53.5%)	154 (60.2%)			
Breastfeeding duration: months	6.42 (5.88) (n = 251)	7.79 (5.91) (n = 251)	*F* (1,494) = 3.21	0.074	Partial η^2^ = 0.01
Number of doctor appointments^b^	6.60 (6.24)	7.33 (5.99)	*F* (1,504) = 2.94	0.087	Partial η^2^ = 0.01
Number of vaccinations^b^^,^^c^	7.93 (1.89)	7.66 (2.05)	*F* (1,504) = 1.79	0.181	Partial η^2^ = 0.00
Risk behavior score^b^^,^^d^	0.65 (0.61)	0.85 (0.72)	*F* (1,504) = 8.36	**0.004**	Partial η^2^ = 0.02

CCS, childhood cancer survivors.

a “How fearful are you that your child might develop cancer?,” marked on a 10 cm long scale.

b Covariates: age and sex of parent and child, country, and highest vocational education in household.

c Vaccinations: measles, mumps, rubella, diphtheria, tetanus, pertussis, poliomyelitis, hepatitis B, and haemophilus influenzae type B.

d Sum score of the number of behaviors seen as risky by CCS: alcohol and nicotine consumption during pregnancy, smoking at home in child’s presence, and breastfeeding duration < 6 6 months. Significant *p* values are marked in bold.

When comparing different levels of fear and preventive behavior (number of doctor appointments and vaccinations, risk behavior score) within types of cancer (hematologic tumors, brain tumors, and other solid tumors) in CCS, a MANCOVA with the covariates age when treated and time since treatment yielded no significant results (*F* (8,498) = 0.77, *p* = 0.628) and did not fulfill all necessary requirements, as the Box test was significant.

### 3.3 Correlational analyses between fear and behavior

Partial correlations with the covariates age and sex of parent and child, country, highest vocational education in household, and type of cancer as well as age when treated in CCS yielded negative correlations between fear and the number of children in both groups as well as between fear and the time since therapy after the employment of FDR (see Table 4). Moreover, the number of doctor appointments correlated positively with fear for siblings, but not for CCS. When calculating correlations between fear and the three age variables (current age of parent and child, age of parent when treated), each variable was not used as a covariate in the corresponding analysis. A negative correlation between fear and the parental age was found in CCS, but not in siblings. Other correlations between fear and age did not remain statistically significant after FDR. Fisher’s r-to-z transformation was employed, and the following comparison of CCS and siblings’ correlations showed no significant group differences.

**Table 4:** Partial correlations between fear, age, number of children as well as preventive behavior in CCS and siblings (*n* = 512).

Variables	Groups				Statistics	
	CCS (*n* = 256)		Siblings (*n* = 256)			
	*r*	*p*	*r*	*p*	Fisher’s *z*	*p*
Age of parent (current)^a^	−0.16^*^	**0.014**	−0.03	0.613	1.48	0.140
Age of parent (at oncological therapy)^b^	0.13^*^	0.047				
Age of child (current)^c^	−0.09	0.161	−0.13^*^	0.039	0.87	0.385
Time since therapy^d^	−0.19^**^	**0.003**				
Number of children^d^	−0.21^**^	**0.001**	−0.16^**^	**0.009**	0.58	0.560
Number of doctors appointments^d^	0.09	0.169	0.19^**^	**0.003**	1.15	0.251
Number of vaccinations^d^	0.06	0.319	0.07	0.549	0.11	0.910
Risk behavior score^e^	−0.05	0.480	−0.06	0.330	0.11	0.910

CCS, childhood cancer survivors, Fear: “How worried are you that your child might develop cancer?,” marked on a 10 cm long scale. Results remaining significant after the employment of false discovery rate are marked in bold. ^*^p < 0.05, ^**^p < 0.01, ^***^p < 0.001.

a Covariates: sex of parent and child, age of child, country, highest vocational education in household, and type of cancer as well as age when treated.

b Covariates: sex and age of parent and child, country, highest vocational education in household, and type of cancer in CCS.

c Covariates: sex of parent and child, age of parent, country, highest vocational education in household, and type of cancer as well as age when treated.

d Covariates: sex and age of parent and child, country, highest vocational education in household, and type of cancer as well as age when treated in CCS; Vaccinations: measles, mumps, rubella, diphtheria, tetanus, pertussis, poliomyelitis, hepatitis B, and haemophilus influenzae type B.

e Sum score of the number of favorable and unfavorable health-related behaviors: alcohol and nicotine intake during pregnancy, smoking at home in child’s presence, and breastfeeding < 6 months.

## 4 Discussion

In this explorative multicentric study, we used a homogenous data set of 512 age- and sex-matched adult CCS and siblings of CCS (256 in each group) to assess the level of fear that offspring could develop cancer and associated parental preventive and health behavior. As hypothesized, CCS reported higher levels of fear than the siblings. With increasing age as well as with increasing number of children, levels of fear decreased in CCS. The number of doctor appointments correlated with fear only in siblings.

The finding that CCS had higher levels of fear regarding a potential development of cancer in their offspring is in line with findings demonstrating higher levels of anxiety in CCS compared to the general population (Michel et al., 2010), although the effect size was very small. Nevertheless, after having survived cancer at a young age, it is not surprising that CCS anxiety and fear levels are higher, as they themselves were affected and often suffer long-term consequences of a life-threatening disease and its intensive treatment. However, this heightened fear does not stem from fact, as offspring of CCS are as healthy as other children (Sommerhäuser et al., 2021; Borgmann-Staudt et al., 2022). Rather, these results indicate that survivors harbor dysfunctional beliefs that their offspring have a higher risk of developing childhood cancer, which should be addressed during therapy. Siblings, who were exposed to the threat of cancer as well, were less fearful than CCS; this may indicate that indirect exposure to cancer and its treatment influenced their parenting differently. As siblings of CCS, their experience of cancer at a young age is different to their affected siblings, but it might also differ from the general population. It therefore would have been interesting to include a third group of healthy controls with no exposure to cancer whatsoever. Additionally, it would be interesting to investigate the fear and anxiety levels of CCS who have chosen to remain childless (Hohmann et al., 2011).

Mothers of children in CCS families consumed less alcohol and nicotine in pregnancy than in siblings’ families. Being more concerned that their children might develop cancer, CCS may exhibit a heightened sense of caution regarding their lifestyle during pregnancy or the lifestyle of their spouses when pregnant. This increased vigilance could be aimed at minimizing potential risks for their offspring. Although it is unclear whether the difference in substance use remains the same before and after pregnancy, CCS seem to generally display more health-conscious behavior in themselves or within the family, as shown by other studies finding alcohol and nicotine consumption of CCS to be reduced in comparison to siblings. Surviving childhood cancer may have heightened their awareness and appreciation of the importance of good health, although small effect sizes were reported in the current study as well as others (Klosky et al., 2012; Marjerrison et al., 2016).

Breastfeeding is known to have a variety of beneficial short- and long-term health effects both for the lactating mother as well as the breastfed infant; this includes the promotion of child development, the protection against disease, and infant mortality reduction (Gertosio et al., 2016). Many of these effects are even more pronounced if infants are breastfed for a minimum of four to 6 months. Hence, breastfeeding for at least 6 months can be viewed as an important component of healthy parenting behavior, a focal point of our study. When comparing both cohorts, fewer CCS children were breastfed. The duration of breastfeeding was comparable, and approximately 50% of children in CCS and siblings’ families were breastfed for at least 6 months. Generally, most CCS women are equally able to breastfeed compared to the general population, however, a subgroup of CCS may experience difficulties in lactation, especially after chest/thoracic irradiation. A previous study found that fewer CCS women planned to breastfeed than healthy controls, as they feared lactation insufficiency and had misconceptions about cancer being passed through breastmilk (Ogg et al., 2020). The current study’s results, especially the small to moderate effect size (despite the inclusion of CCS men in the analysis), might hint at an underlying belief about negative effects of breastfeeding (at least among CCS women) as well, highlighting the importance of providing CCS women with supportive and extensive information aimed at encouraging breastfeeding. More research in this area is warranted.

Interestingly, the most influential age variable was parental age, which was negatively related to fear in CCS, but not in siblings. Neither survivor age when treated nor their child’s current age was as influential, showing that the mere progression of time seems to be enough to reduce fear. This is reinforced by the negative correlation between fear and the time since oncological therapy. Perhaps, temporal distance also helps CCS mentally distance themselves from their anxiety and past suffering, to process their experiences, and become more adept at developing coping strategies. Fittingly, siblings, who do not have this significant time of suffering from cancer to overcome, show no association between fear and age. The lack of influence of their own age during therapy on CCS’ fear reinforces the understanding that surviving cancer during childhood is a highly stressful and traumatic experience at any age. Interestingly, as the number of children increased, overall fear in both CCS and siblings decreased. Apparently, experiencing the cancer-free development of one or more of their children seemed to curb the initial fear reported by CCS, and may have resulted in a more positive attitude towards the health of additional children. This might also contribute to a heightened self-efficacy, reinforcing preventive and favorable behavior, which might further reduce fear. In turn, both CCS and siblings with lower levels of fear might feel encouraged to have more children. Considering this, we would like to reiterate and reinforce the importance of educating both groups about the nonexistent increased cancer risk for their offspring.

In contrast to siblings, fear did not correlate with the number of doctor appointments in CCS. Perhaps, by enduring extreme suffering during childhood, CCS experienced more self-efficacy by learning how to cope with their fear independently. As survivors not only trusted the healthcare system, but also understand its limitations, they may put greater emphasis on their own agency to prevent cancer development. Moreover, by gaining expertise through their own disease and treatment, they might be more competent in deciding the necessity of a doctor appointment as well as the behavior contributing to a healthy life. Another possibility is knowing that doctor appointments would not alleviate their fear, CCS may have found other reliable sources of comfort.

This study has several limitations. Firstly, due to the cross-sectional design of the study, we refrained from making any causal inferences. Furthermore, since the study was not pre-registered, certain sections must be interpreted as exploratory evidence. Secondly, it was not possible to include the subjective state of children’s health and the objective assessment of children’s health due to missing data. Accordingly, the duration of breastfeeding was possibly influenced by circumstances that were not sufficiently assessed in the current study, such as irradiation during cancer treatment. Furthermore, instead of the number of recommended medical check-ups, the number of total doctor appointments was included. Thirdly, CCS and siblings differed in highest vocational education and country, both of which were used as control variables in statistical analyses. It would have been better to include education as a matching variable, which was not possible due to lack of data. Fourthly, another factor that was not included and might have influenced the results, especially fear, was sibling order. Older siblings might have been more affected during CCS diagnosis and treatment, while younger siblings might have been too young to understand the severity of the situation, subsequently developing different levels of fear response. Fifthly, the variable fear was a single item with 0 = little–no fear and 10 = highly fearful, with values in-between being highly subjective and inter-individually different. Moreover, language differences may have led to differences in the understanding of fear. Sixthly, the item assessing fear as well as the risk behavior score were not standardized and remain to be validated. It is suggested to use valid and reliable measurement instruments to assess these constructs in future studies. Seventy, although we hypothesized that the type of cancer influences fear about offspring and health and parenting behavior, this research question could not be answered due to a lack of statistical preconditions. Lastly, given the increased risk of cancer in siblings of CCS (Friedman et al., 2005), who also grow up with highly distressed parents (Ljungman et al., 2014), and for whom the issue of childhood cancer is very real, it might be reasonable to survey people who are unaffected by this topic as an additional control group in future studies. To offer adequate patient counseling and address concerns, further assessment of offspring health is crucial.

In summary, although effect sizes throughout the study are rather small, our observations indicate that as CCS age and the time since end of therapy increases, the fear that their offspring might experience health impairment appears to diminish. Additionally, survivors who observe the cancer-free development of their own children seem comforted and encouraged by the self-efficacy gained through practicing preventive behaviors. In contrast, siblings seem to place more reliance on the authority of the healthcare system and attach less importance to preventive behavior. It is important to educate both groups regarding the misperception of their offspring’s increased risk for health impairments due to their own experience with cancer. This awareness can contribute to a more accurate understanding and informed decision-making regarding preventive measures for the well-being of their children.

## Data availability statement

The raw data supporting the conclusions of this article will be made available by the authors, without undue reservation.

## Ethics statement

The studies involving humans were approved by Charité-Universitätsmedizin Berlin. The studies were conducted in accordance with the local legislation and institutional requirements. The participants provided their written informed consent to participate in this study.

## Author contributions

ND: Conceptualization, Formal analysis, Investigation, Methodology, Project administration, Resources, Supervision, Writing – original draft, Writing – review & editing. EF: Data curation, Formal analysis, Investigation, Resources, Software, Validation, Visualization, Writing – original draft, Writing – review & editing. AB-S: Conceptualization, Validation, Writing – review & editing. CF: Writing – review & editing. SK-B: Writing – review & editing. KK: Conceptualization, Writing – review & editing. JK: Conceptualization, Writing – review & editing. HL: Conceptualization, Writing – review & editing. GM: Writing – review & editing. AM: Writing – review & editing. EN: Writing – review & editing. AP: Conceptualization, Writing – review & editing. MT: Writing – review & editing. ER: Writing – review & editing. KW: Writing – review & editing. MB: Formal analysis, Funding acquisition, Methodology, Project administration, Resources, Supervision, Validation, Writing – review & editing.
